# Commentary: Revised contraindications for the use of non-medical WB-electromyostimulation. Evidence-based German consensus recommendations

**DOI:** 10.3389/fspor.2024.1425233

**Published:** 2024-07-22

**Authors:** Dejan Reljic, Hans Joachim Herrmann, Yurdagül Zopf

**Affiliations:** ^1^ Hector-Center for Nutrition, Exercise and Sports, Department of Medicine 1—Gastroenterology, Pneumonology and Endocrinology, University Hospital Erlangen, Friedrich-Alexander University Erlangen-Nürnberg, Erlangen, Germany; ^2^ Department of Medicine 1—Gastroenterology, Pneumonology and Endocrinology, University Hospital Erlangen, Friedrich-Alexander University Erlangen-Nürnberg, Erlangen, Germany

**Keywords:** whole-body electromyostimulation, exercise oncology, cancer, tumor diseases, exercise safety, contraindications

**A Commentary on**
Revised contraindications for the use of non-medical WB-electromyostimulation. Evidence-based German consensus recommendations By von Stengel S, Fröhlich M, Ludwig O, Eifler C, Berger J, Kleinöder H, et al. (2024). Front Sports Act Living. 6:1371723. doi: 10.3389/fspor.2024.1371723.

With great interest, we read the article by von Stengel et al. (1). This updated review provides valuable information on the safe and effective use of non-medical whole-body electromyostimulation (WB-EMS). As one of the leading groups in applying WB-EMS in chronic disease cohorts, we would like to comment on a few issues. Due to the limited possible scope, we focus our remarks on the use of WB-EMS in cancer patients.

The authors undertook a comprehensive revision of their previously published guidelines (2) and one of the significant modifications they made was the reclassification of cancer/tumor diseases from absolute to relative contraindications. Although the authors noted that there was intense discussion about completely removing cancer/tumor diseases from the contraindications catalog, these disorders remained in the list due to “*an ongoing disagreement*”. In this regard, we acknowledge the authors' cautiousness when it comes to assessing the risk/benefit-ratio of using WB-EMS in oncology settings. However, based on current evidence and our extensive clinical experience with the application of WB-EMS in cancer patients, we strongly suggest that some aspects need to be reconsidered before adopting this conclusion and that the consensus recommendations would benefit from some revisions.

In general, exercise recommendations for cancer patients have evolved significantly in the last decades. While until the late 1990s, it was typically advised to rest and avoid physical activity during cancer disease, targeted exercise is now recognized as an important cornerstone in multimodal oncological treatment (3). This paradigm shift is based on a steadily growing body of research documenting the multiple benefits of physical exercise during cancer, including, for example, reduced fatigue (4–6) improved physical function (4–6), quality of life (4–6) and side effects management (4–6) as well as decreased risk of disease recurrence (5, 6) and death (5, 6). In recent years, a number of studies have expanded the knowledge in this field by evaluating the feasibility and efficacy of novel exercise interventions with different intensities, durations, and modes in cancer populations. As a result, exercise types, such as eccentric resistance training (7), blood flow restriction training (7) or high-intensity interval training (8) have been started to be considered to be safe and effective for application in oncology settings.

As part of this process, our research group has played a pioneering role in investigating the use of WB-EMS in cancer patients. Our studies have demonstrated that WB-EMS can be regarded a valuable exercise method to counteract muscle wasting and improve measures of physical functioning in patients with different types and stages of cancer (9–11). For instance, we found that 12 weeks of WB-EMS, performed twice weekly for 12 weeks, resulted in an average 0.53 kg higher skeletal muscle mass in advanced cancer patients compared to inactive control patients (10), which can be considered clinically meaningful. Moreover, WB-EMS exercise translated into improved gait velocity and 6-min walking distance in a clinical trial involving patients with advanced cancer (9). From a practical point of view, it should be noted that particularly physically weak patients who are unable to perform adequate exercise training and those with certain physical impairments, such as fracture risk bone metastases, may benefit from WB-EMS as it offers a time-efficient and joint-friendly option to conventional resistance training with free weights or weight machines. In this context, we would like to highlight that WB-EMS has now been successfully used at our Center for almost 10 years in different studies and exercise programs for cancer patients without any recorded serious adverse events. Additionally, it is noteworthy that the literature (12–15) has not defined any age restrictions for the use of WB-EMS in adults, rendering it a viable exercise option for older patients.

Importantly, to date there is not a single indication that WB-EMS exercise is associated with any specific harmful effects on cancer disease progression, such as, for example, accelerated tumor growth, or general disease exacerbation. In contrast, two *in vitro* studies from our group provided promising preliminary results indicating that muscle building through WB-EMS may have an inhibitory effect on human cancer cell proliferation and viability (16, 17). In these studies, the serum from advanced-stage cancer patients with prostate or colorectal (16) and pancreatic cancer (17) was used to incubate cancer cell cultures and it was a remarkable finding that cell growth was significantly hampered, pointing to an anti-tumor effect of myokines released from the muscles stimulated by WB-EMS. Therefore, taking into account the available scientific evidence, we would like to point on the following three issues.

First, it is important to recognize that the physical abilities and limitations of cancer patients can vary widely depending on the type and stage of cancer, their overall health status, and their specific treatment. In daily clinical practice, we observe a broad spectrum of exercise tolerance among cancer patients, ranging from individuals with significant physical impairments to those without limitations and restrictions for any type of training. Consequently, listing cancer/tumor disease as a general relative contraindication for WB-EMS is too unspecific and implies a cancer-specific risk associated with this type of exercise, which is not supported by current evidence. Instead, we recommend that the present consensus guidelines align with the general safety protocols for exercise in cancer patients and adopt the contraindications defined therein (18, 19). More specifically, the absolute contraindications for exercise in cancer patients are similar to those for other chronic diseases and the cancer-related relative contraindications include anemia, thrombocytopenia, bone metastases, accompanying cardiovascular diseases or symptoms, administration of cytostatic agents on the same day, mediastinal/cardiac radiation therapy, and flu-like symptoms during immunotherapy (18, 19). Based on our practical experience, we would add two more conditions where WB-EMS should be carefully monitored and/or adjusted in oncology settings: an increased risk of lymphedema following lymph node removal and unhealed scars after surgery. However, these conditions do not represent contraindications *per se*, as WB-EMS can be adapted by omitting electrical stimulation on certain body parts (e.g., the abdomen).

Second, we acknowledge that the current consensus recommendations specifically address non-medical (commercial) WB-EMS, which differs from medical WB-EMS. By definition, medical WB-EMS training is “*(1) a primarily therapeutic intervention, (2) based on an existing diagnosis, (3) provided by qualified therapeutic personnel, (4) in compliance with current guidelines and, (5) using medical devices*” (13). In this context, we strongly advocate that cancer patients with relative contraindications for exercise (18, 19) should exclusively perform WB-EMS (as well as other types of exercise) in well-controlled medical settings. For these patients, we additionally recommend close monitoring of muscle damage markers, such as creatine kinase (CK) blood concentrations, especially during the initial weeks of exercise, to avoid muscular overloading. However, for patients without established contraindications for exercise (18, 19), we propose that WB-EMS training can be considered under less stringent conditions, provided that two criteria are met: (1) as with any other exercise intervention in chronic disease cohorts, a thorough medical examination should be conducted beforehand to rule out any general contraindications for exercise and to assess the patient's individual physical capacity; and (2) WB-EMS should only be administered by qualified sports- or physiotherapists who are trained in the use of this exercise method.

Third, we note that our two aforementioned recommended prerequisites for using WB-EMS in non-medical settings (i.e., medical clearance and qualified supervision) were used by the authors as criteria to define relative contraindications for WB-EMS. However, this approach is somewhat misleading as it suggests that WB-EMS generally constitutes a relative contraindication for all individuals who are advised to undergo a medical examination before engaging in exercise according to current guidelines (20). This includes, for example, apparently healthy adults who wish to start exercising for the first time or who want to return to sport after a prolonged period of physical inactivity.

In conclusion, we recommend the authors to reconsider their definition of relative contraindications for the use of WB-EMS. For cancer patients specifically, we suggest that WB-EMS should not be broadly categorized as a relative contraindication but rather tailored to the individual health status based on general exercise oncology guidelines. Finally, as a practical message, we encourage health professionals working with cancer patients to incorporate WB-EMS into oncology exercise programs—provided that patients have undergone proper medical clarification and that training is supervised by qualified personnel. WB-EMS is certainly not a “magic exercise bullet” but it can be a useful and safe tool for clinical exercise programs, including oncology settings.
